# The Selective Target of Capsaicin on FASN Expression and De Novo Fatty Acid Synthesis Mediated through ROS Generation Triggers Apoptosis in HepG2 Cells

**DOI:** 10.1371/journal.pone.0107842

**Published:** 2014-09-25

**Authors:** Hathaichanok Impheng, Sutatip Pongcharoen, Lysiane Richert, Dumrongsak Pekthong, Piyarat Srisawang

**Affiliations:** 1 Department of Physiology, Faculty of Medical Science, Naresuan University, Phitsanulok, Thailand; 2 Department of Medicine, Faculty of Medicine, Naresuan University, Phitsanulok, Thailand; 3 Laboratoire de Toxicologie Cellulaire, Faculté de Médecine et de Pharmacie, Université de Franche-Comté, Besançon, France; 4 Department of Pharmacy Practice, Faculty of Pharmaceutical Sciences, Naresuan University, Phitsanulok, Thailand; Wayne State University, United States of America

## Abstract

The inhibition of the mammalian *de novo* synthesis of long-chain saturated fatty acids (LCFAs) by blocking the fatty acid synthase (FASN) enzyme activity in tumor cells that overexpress FASN can promote apoptosis, without apparent cytotoxic to non-tumor cells. The present study aimed to focus on the potent inhibitory effect of capsaicin on the fatty acid synthesis pathway inducing apoptosis of capsaicin in HepG2 cells. The use of capsaicin as a source for a new FASN inhibitor will provide new insight into its possible application as a selective anti-cancer therapy. The present findings showed that capsaicin promoted apoptosis as well as cell cycle arrest in the G0/G1 phase. The onset of apoptosis was correlated with a dissipation of mitochondrial membrane potential (ΔΨm). Apoptotic induction by capsaicin was mediated by inhibition of FASN protein expression which was accompanied by decreasing its activity on the *de novo* fatty acid synthesis. The expression of FASN was higher in HepG2 cells than in normal hepatocytes that were resistant to undergoing apoptosis following capsaicin administration. Moreover, the inhibitory effect of capsaicin on FASN expression and activity was found to be mediated by an increase of intracellular reactive oxygen species (ROS) generation. Treatment of HepG2 cells with capsaicin failed to alter ACC and ACLY protein expression, suggesting ACC and ACLY might not be the specific targets of capsaicin to induce apoptosis. An accumulation of malonyl-CoA level following FASN inhibition represented a major cause of mitochondrial-dependent apoptotic induction instead of deprivation of fatty acid *per se*. Here, we also obtained similar results with C75 that exhibited apoptosis induction by reducing the levels of fatty acid without any change in the abundance of FASN expression along with increasing ROS production. Collectively, our results provide novel evidence that capsaicin exhibits a potent anti-cancer property by targeting FASN protein in HepG2 cells.

## Introduction

Capsaicin (*trans*-8-methyl-*N*-vanillyl-6-non-enamide) is the major component in hot chili peppers and several types of red peppers of the genus *Capsicum*. It constitutes approximately 40–60% of the six natural capsaiciniod contents of this herb [1], [2]. In particular, it is commonly and frequently consumed worldwide as a spice and food additive as well as a drug for traditional medications. Over the past decades, research studies have demonstrated the applications of capsaicin as a specific and potent anti-carcinogenic agent through the apoptosis pathway in both in vitro and in vivo cancer models, whereas it does not induce cytotoxicity in normal cells [3], [4], [5], [6], [7]. The mechanisms underlying capsaicin-induced apoptosis may involve multiple signaling pathways and depend upon cell types [3], [8]. Capsaicin acts as the high affinity agonist that directly binds to the transient receptor potential vanilloid subtype 1 (TRPV1) and TRPV6 receptors that belong to the transient receptor potential (TRP) superfamily of nonselective cation-channels. They are overexpressed mainly in human cancer cells compared to normal cells [4], [5], [8], [9], [10], [11], including HepG2 cells [12]. The binding of capsaicin to TRPV1 induces apoptosis via multiple mechanisms, including the generation of reactive oxygen species (ROS), such as superoxide radicals and hydrogen peroxide as well as impairment of ER calcium homeostasis. In consequence, this leads to the reduction of mitochondrial transmembrane potential (ΔΨm), activation of pro-apoptotic Bax, suppression of anti-apoptotic Bcl-2, and induction downstream caspase-3-activated apoptosis pathway in several cancer cells [11], [13]. Similarly, binding of capsaicin to TRPV6 triggers apoptosis via an activation of excessive calcium influx that leads to stimulating the apoptotic regulating factor, calpains, the calcium-dependent intracellular cysteine proteases in human small cell lung cancer (SCLC) [5].

Besides the TRPV-1 activation pathway, Skrzypski et al., 2014 have suggested that a TRPV1-independent pathway contributes to the cytotoxic effect of capsaicin in pancreatic BON cells through blocking of complexes I and III of the respiratory chain that leads to impairment of ΔΨm [4]. Capsaicin-induced apoptosis is also mediated by ROS overproduction generated from the inhibiting of the plasma membrane NADH-oxidoreductase (PMOR) electron transport chain [14], [15]. This inhibition results in the disturbance of cellular redox homeostasis and also induces damage of the redox sensitive proteins and lipids, leading to disruption of ΔΨm and apoptosis in cancer cells [6], [16], [17]. Taken together, capsaicin is accepted as offering a potentially novel anti-cancer therapy.

Several recent studies have suggested that suppression of the expression or activities of enzymes that participate in the synthesis of the *de novo* fatty acid in cancer cells provides a novel therapeutic approach causing cell cytotoxicity and cell death by means of apoptosis [18], [19], [20]. It has been reported that supplementing cells with palmitate, stearate, or oleate ameliorates the fatty acid depletion-induced cytotoxic effect in cancer cells, suggesting an important role of the *de novo* synthesis of fatty acid for cancer cell viability [21]. The pharmacological anti-cancer agents, including cerulenin, C75, triclosan, and orlistat, have been extensively evaluated in various cancer cells to exert apoptosis through anti-fatty acid synthesis activity [22]. Besides the use of pharmacological fatty acid synthesis inhibitors as anti-cancer drugs, the mechanism of capsaicin-induced apoptosis via targeting the *de novo* fatty acid synthesis inhibition will provide a new perspective benefit to suppress cancer.

Due to diminution of vascular supply and deprivation of the nutritional microenvironment, cancer cells up-regulate the hypoxia inducible factors (HIFs) to control the expression of transformed genes of glycolysis and OXPHOS pathways [23]. This leads to induction of the cellular ATP-generating system to be not exclusively dependent on mitochondrial oxidative phosphorylation (OXPHOS) but to concomitantly rely on anaerobic metabolism of glucose regardless of the presence of an oxygen supply [24]. These features of enzyme expression reduce the requirements of oxygen for ATP production through OXPHOS and switch the generation of ATP from OXPHOS to glycolysis [25], [26]. In addition to the alteration of the metabolic pathway, the translocation of the carbons from OXPHOS for the *de novo* synthesis of saturated long-chain fatty acids (LCFAs) becomes predominant for controlling the cellular function via β–oxidation [27]. In untransformed cells, OXPHOS contributes to 70% of the ATP-generating metabolism while fatty acid synthesis is exclusively generated from exogenous transported fatty acids derived from nutritional consumption. It has been reported that enzymes responsible for this *de novo* lipogenesis pathway are highly expressed in cancer cells [26]. Fatty acid synthase (FASN), one of the important lipogenic enzymes, catalyzes the synthesis of LCFAs from substrates, acetyl-CoA, malonyl-CoA, and a reducing agent NADPH. The most abundant *de novo* LCFAs is palmitatic acid. The expression of FASN and its activity are undetectable in most normal tissue. In addition to cancer cells, high expression of FASN has been reported in lipogenic tissue, such as the liver [28]. The abundant expression of FASN and its function on *de novo* fatty acid synthesis in cancer cells is accompanied by carcinogenesis and is relevance to unsatisfactory prognosis [29]. Several studies have demonstrated that suppression of FASN activity promotes apoptosis in cancer cells. However, the inhibition of FASN is unable to suppress proliferation of normal cells that have low levels of FASN expression. This suggests that the *de novo* synthesis of LCFAs by inhibition of FASN in cancer cells becomes a focus for the selective target of anti-cancer therapeutics [30], [31], [32], [33]. The biological mechanisms of apoptosis induction by inhibition of FASN and fatty acid synthesis has been reported to be due to the lack of the end product LCFA *per se*, or the downstream toxic effects of an increase of the malonyl-CoA synthesis pathway [22].

In addition to the synthetic *de novo* fatty acid synthesis inhibitors having an impact for the treatment of cancer, the demonstration of natural dietary compounds that have the ability to inhibit fatty acid synthesis and suppress the growth of cancers could promote a potential therapy for this disease. Research studies of dietary phenolic compounds, such as quercetin have been reported to induce apoptosis in HepG2 cells through downregulation of FASN expression and its activity on intracellular fatty acid synthesis [33]. Resveratrol is found to suppress proliferation of cancer stem-like cells isolated from breast cancer cells through suppression of FAS expression [32]. Piperine shows apoptotic induction via downregulation of *FAS* gene expression in breast cancer cells [31]. In addition to anti-FASN activity, (−)-epigallocatechin-3-gallate (EGCG) has been shown to suppress the growth and induce apoptosis of lung cancer without producing an undesirable effect on carnitine palmitoyl transferase I (CPT-1) and β-oxidation activities which result in a severe decrease of body weight as reported in C75-treated animals [34]. Thus, we propose to continue the open perspective hypothesis that capsaicin can become one of the FASN inhibitors that will find an ultimate clinical application in treating cancer patients. Therefore, the present study was undertaken to focus and investigate the inhibitory effect of capsaicin on FASN expression and its fatty acid synthesis activity to induce apoptosis via the mitochondrial dependent pathway in HepG2 cells. The selective target of capsaicin on fatty acid synthesis was assessed with HepG2 cells compared to the human hepatocytes. This study demonstrates the selective anti-cancer effect of capsaicin on HepG2 cells that express higher levels of FASN than normal cells. Capsaicin-induced mitochondrial dependent apoptosis in HepG2 cells is obviously mediated by ROS generation that promotes a downstream inhibitory effect on the *de novo* fatty acid synthesis pathway. Increment of ROS generation and disruption of ΔΨm confer proapoptotic action of capsaicin in HepG2 cells. The consequent accumulation of malonyl-CoA following deprivation of fatty acid level appears to be a major causative of capsaicin-induced apoptosis in HepG2 cells. Here, our present work strongly suggests that the apoptotic mechanism of capsaicin provides a novel therapeutic intervention as a selective anti-cancer agent that potentially targets the lipogenic enzyme FASN in HepG2 cells.

## Materials and Methods

### Chemicals and reagents

Capsaicin and C75 were obtained from Sigma Chemical Co. (St. Louise, MO, USA). Eagle's Minimum Essential Medium (EMEM), phosphate-buffered saline (PBS), fetal bovine serum (FBS), penicillin-streptomycin, trypsin-EDTA, sodium pyruvate, L-glutamine, sodium bicarbonate solution, non-essential amino acid solution, and PBS tablets were obtained from Gibco BRL (Grand Island, NY, USA). Propidium iodide molecular probe were purchased from Life Technology (Invitrogen, Grand Island, NY, USA). M-PER mammalian protein extraction reagent, BCA protein assay reagent (bicinchoninic acid), polyvinylidenedifluoride membranes (PVDF) membrane, and Halt Protease Inhibitor Cocktail were purchased from Thermo Scientific (Rockford, IL, USA). Anti-fatty acid synthase (FASN) and anti-β-actin antibodies were purchased from Abcam (Biomed Diagnostics (TH) Co., Ltd, Thailand). Anti-ACC and anti-ACLY were purchased from Merck Millipore (Darmstadt, Germany) and Cell Signaling Technology Inc (Boston, MA, USA), respectively. All other chemicals and reagents were the highest grade available.

### Cell culture

HepG2 cell line was purchased from American Type Culture Collection (ATCC), ATCC number: HB-8065, (Manassas, VA, USA). HepG2 cells were cultured in EMEM media containing 10% FBS and 1% penicillin-streptomycin (100 units/mL of penicillin and 10 µg/mL of streptomycin) at 37°C in a 5% CO_2_ humidifier incubator. The medium was changed every 2 days and confluent cultures were passaged by trypsinization. Primary human hepatocytes were kindly provided by Prof. Dr. Lysiane Richert, Scientific Director KaLy-Cell, 20A, rue du Général Leclerc, 67115 Plobsheim, France. Cells were cultured in Human Hepatocyte Maintenance Medium (Primacyt, Schwerin, Germany) containing 0.1 M DEX, 50 mg/l gentamicin, 4 mg/l insulin, and 5% fetal calf serum. Cells were allowed to attach by incubating under a CO_2_/air (5∶95%) humidified atmosphere maintained at 37°C as described previously [35], [36].

### Determination of the effect of capsaicin on cell viability and proliferation

The antiproliferative effect of capsaicin on HepG2 cells was determined by measuring cell viability using MTT assay. Briefly, cells were seeded into 96-well plates (SPL Life Sciences Inc., Korea) at a density of 1×10^4^ cells/well. Capsaicin was freshly prepared by dissolving it in DMSO, and diluting it with completed growth EMEM medium to give final concentrations at 0.05 to 2 mM with DMSO at 0.1%. Cells were allowed to attach overnight before they were incubated with different concentrations of capsaicin for 24 or 48 h. The control samples were cultured in EMEM medium only and in 0.1% DMSO in a complete EMEM medium. After treatments, 100 µL of MTT solution (5 mg/mL in PBS) was added to each well and incubated for 2 h at 37°C. Then, the solution was removed, the formazan was dissolved in 150 µL of DMSO and measured spectrophotometrically at 540 nm using Synergy HT Microplate Reader (BioTek Instruments, Inc, Winooski, VT, USA). The percentage of cell viability was calculated in comparison with the control group. The experiments were performed on at least three different occasions and triplicate wells were analyzed for each experiment. The IC_50_ of capsaicin was defined as the concentration of capsaicin that caused a 50% reduction in cell viability compared with the control. IC_50_ was calculated by linear regression by plotting the percentage of cell viability versus capsaicin concentration using Graph Pad Prism version 5.

### Analysis of the effect of capsaicin on cell death induction through apoptosis

Apoptosis was determined by flow cytometry using a double staining protocol: Annexin and PI dyes. The Annexin V staining was used to assess the early stage of apoptosis with the externalization of phosphatidylserine (PS) while the cells stained with both Annexin and PI were defined as the late apoptotic cells. Approximately 1×10^6^ cells of HepG2 cells were cultured in a 60 mm^3^ petri dish and allowed to attach overnight. Then, cells were incubated with capsaicin at a concentration range that covered the logarithmic phase of the concentration-response (% cell viability) curve shown in the MTT assay for 24 and 48 h. Cells without capsaicin and with 0.1% DMSO were defined as the control. After treatment, both adherent and floating cells were harvested, centrifuged, re-suspended in Annexin binding buffer and then stained with Alexa Fluor 488 annexin V and PI according to the protocol of the Alexa Fluor 488 annexin V/Dead Cell Apoptosis Kit (Life Technology, Invitrogen). After 15 minutes, cells were analyzed by a FACScalibur flow cytometry (Becton Dickinson (BD), Franklin Lakes, New Jersey, USA) and the data were analyzed using CellQuestPro software (BD). The analysis of apoptosis was performed in five independent experiments.

### Determination of the effect of capsaicin on nuclear morphology alteration of apoptotic cells

One of the specific characteristics of apoptotic cells is alterations of nuclear morphology, such as condensation of chromatin and dissociation of DNA fragmentations [3]. DAPI staining was used to determine the changes of DNA during the apoptotic process. Approximately 1×10^6^ cells of HepG2 cells were cultured in a 60 mm^3^ cell culture dish and allowed to attach overnight. The cells were incubated with capsaicin at 0.05 mM for 0–48 h. Cells without capsaicin were defined as the control. The effect of DMSO on cell apoptosis was also examined. After incubation periods, cells were harvested, washed with PBS, and then fixed overnight with 70% ethyl alcohol. The fixed cells were then stained with 1 mg/mL 4′, 6′-diamidino-2-phenylindole (DAPI) for 30 min in the dark at room temperature. The stained cells were detected with a fluorescent microscope at 40x magnification. The analysis of nuclear morphology was performed in three independent experiments.

### Determination of the effect of capsaicin on cell cycle regulation

HepG2 cells were cultured in a 60 mm^3^ petri dish at the seeding density of 1×10^6^ cells. After an overnight period of attachment, cells were treated with different concentrations of capsaicin (0.05 to 1 mM) for 24 and 48 h. Then, the treated and vehicle control cells were harvested and fixed with pre-cooled 70% ethanol before being stored at 4°C for 24 h. The fixed cells were then incubated with 100 µg/mL of RNase A at 37°C for 30 minutes. After the incubation period, cells were then stained with 20 µg/mL of PI for 2.5 h in the dark at room temperature. Cellular DNA content was analyzed by FACScalibur flow cytometry and at least 2.5×10^4^ events were recorded using CellQuestPro software (BD).

### Measurement of the effect of capsaicin on the disruption of mitochondrial membrane potential (ΔΨm)

The disruption of ΔΨm in HepG2 cells was measured by a FACScalibur flow cytometer using JC-1 Dye-Mitochondrial Membrane Potential Probe (Life Technologies) according to the manufacturer’s suggested protocol. Briefly, HepG2 cells were cultured in a 60 mm^3^ petri dish at a seeding density of 1×10^6^ cells/dish and allowed to attach overnight. Then, cells were incubated with different concentrations of capsaicin ranging from 0.05 to 0.5 mM for 3, 6, 12, and 24 h. Membrane-permeant JC-1 dye was used to determine mitochondrial potential and energized mitochondrial state as indicated by the ratio of red and green fluorescence intensity. The positively charged JC-1 accumulates in the electronegative interior of the mitochondria, forms J-aggregates and consequently exhibits red fluorescence emission at ∼590 nm when mitochondria is in a high polarization state of ΔΨm while this dye exists as a monomer and emits green fluorescence at 530 nm when mitochondria is in a depolarized state. After treatment, all floating and attached cells were harvested and then re-suspended to an approximately 1×10^6^ cells/ml. For the control tube, CCCP 50 µM at the final concentration was added to verify the depolarization of ΔΨm. For the sample tubes, 2 µM of JC-1 dye at final concentration was added to each tube, and they were incubated at 37°C, 5% CO_2_ for 45 minutes. After labeling with JC-1 dye, cells were analyzed by FACScalibur flow cytometry using 488 nm excitation with 530/30 nm and 585/42 nm bandpass emission filters. The data were analyzed using CellQuestPro software (BD).

### Western blotting assay for FASN protein expression

Approximately 1×10^6^ cells of HepG2 cells were plated in a 60 mm^3^ petri dish and allowed an overnight period of attachment. Then, cells were incubated with capsaicin at concentrations ranging from 0.05 to 0.5 mM for 3, 6, 12, 24, and 48 h time periods. After treatment, cells were washed with PBS and re-suspended in M-PER reagent containing 10 µl of concentrated Halt Protease Inhibitor Cocktail per 1 mL of lysis buffer. The supernatant was collected from the lysates after centrifuging at 14,000×g for 10 minutes. Then, proteins were subsequently quantified by BCA protein assay reagent. A total of 7 µg proteins of each sample were heated in SDS sample buffer for 5 min at 95°C. Cytosolic proteins were then separated by 8% SDS-polyacrylamide gel electrophoresis and transferred to PVDF membranes by iBlot 7-Minute Blotting System for 8 minutes (Life Technology, Invitrogen). The membranes were incubated with RAPIDBLOCK SOLUTION for 30 minutes at room temperature. After blocking non-specific binding, membranes were incubated with anti-FASN, anti-ACC, and anti-ACLY antibody at 4°C overnight with 5% skim milk in PBS (1∶1000) and then reacted with horseradish peroxidase-conjugated goat anti-Rabbit IgG secondary antibody (Life technology, Invitrogen) diluted 1∶5000 for 1 h. β-actin was used as an internal standard using a rabbit anti-β-actin and anti-rabbit IgG antibody as a primary and secondary antibody, respectively. The protein bands were developed using the Novex ECL Chemiluminescent Substrate Reagent Kit (Life Technology, Invitrogen). The intensity of the protein bands was determined using a CCD camera (ImageQuant LAS 4000; GE Healthcare Life Sciences, Pittsburgh, PA, USA). Protein expression was quantitated using ImageJ software version 1.46 and presented as a ratio of FASN, ACC, and ACLY band intensity relative to the β-actin band intensity in the same blot (FASN/β-actin [%]).

### Determination of FASN activity

#### Intracellular long chain free fatty acid quantification assay

FASN activity in cellular extracts of HepG2 cells was determined by detection of long chain free fatty acid, the product of the *de novo* fatty acid synthesis pathway, using the Free Fatty Acid Quantification Kit (Abcam) and (BioVision, CA, USA) [22], [37] as described in the manufacturer’s protocol. The principle of this assay was based on the conversion of long chain free fatty acids to CoA derivatives, which were subsequently oxidized. 1×10^6^ HepG2 cells were plated and allowed 24 h of attachment. Capsaicin was incubated at the concentrations of 0.5 and 1 mM for 24 h. Cells were harvested with chloroform-Triton-X 100 (1% Triton-X 100 in pure chloroform). The organic phase solution was collected after centrifugation of the cell lysate samples. The air drying and vacuum drying techniques were used to remove chloroform from the sample. The dried lipid was then dissolved in fatty acid assay buffer. Acyl-CoA synthase was added to the samples and palmitic acid standards to convert to acyl-CoA. The fluorescent probe and the enhancer were subsequently added to the reaction and detected at Ex/Em 535/590 nm using the Synergy HT Microplate Reader (BioTek Instruments). The data were analyzed and expressed in a percentage of intracellular long chain fatty acid compared with the control.

#### Intracellular citrate quantification assay

The important substrate for the *de novo* synthesis of fatty acids is cytosolic citrate which can be quantified by the conversion of citrate to pyruvate via oxaloacetate. The pyruvate product was measured by colorimetric or fluorescence methods. The citrate assay was performed using the Citrate Assay Kit (Abcam) as described in the manufacturer’s protocol. Briefly, HepG2 cells were plated at a density of 1×10^6^ cells and allowed to attach overnight. Cells were incubated with capsaicin at concentrations of 0.5 and 1 mM for 24 h. After treatment, cells were harvested and homogenized with citrate assay buffer and then pelleted by centrifugation. The citrate standard and cell sample were converted to pyruvate, followed by adding the reaction mix containing a fluorescent probe and developer, and further measured fluorescence at Ex/Em535/590 nm using the Synergy HT Microplate Reader (BioTek Instruments) with Gen5 Data Analysis software. Results were expressed as the percentage of intracellular citrate compared with the control.

#### Intracellular triglyceride quantification assay

The concentration of triglycerides in the cells was determined by the Triglyceride Quantification Kit (Abcam) [22]. HepG2 cells were seeded at a density of 1×10^6^ cells/60 mm^3^ petri dish and allowed an overnight period of attachment. Cells were incubated with capsaicin at a concentration of 0.5 and 1 mM for 24 h. After the end of incubation, cells were harvested and homogenized with 5% Triton X-100 in water. The samples were slowly heated from 80–100°C in a water bath for 5 min until the solution turned cloudy. After cooling down to room temperature, the sample was reheated to solubilize all triglycerides and then any insoluble materials were removed by centrifugation. The lipase was added into samples and the triglyceride standard to convert triglycerides to the glycerol and free fatty acid. The fluorescence probe was subsequently added into all standards and samples followed by measurement of the fluorescence intensity at Ex/Em 535/590 nm by using the Synergy HT Microplate Reader (BioTek Instruments) with Gen5 Data Analysis software. Results were expressed as the percentage of intracellular triglycerides compared with the control.

### Measurement of intracellular ROS production

The intracellular reactive oxygen species (ROS) was measured using 5-(and-6)-chloromethyl-2′,7′-dichlorodihydrofluorescein diacetate (CM-H_2_DCFDA; Molecular Probes). CM-H_2_DCFDA is the membrane-permeable fluorescent dye which was taken up into the cells and deacetylated by intracellular esterase to form a non-fluorescent deacetylated H_2_DCF, which was consequently converted to a green fluorescent product DCF by intracellular ROS. Briefly, HepG2 cells were plated at 1×10^6^ cells in a 60 mm^3^ petri dish for an overnight of attachment before being treated with capsaicin at 0.5 mM for 0, 2, 3, and 12 h. After treatment, HepG2 cells were harvested and suspended in PBS containing a final working concentration of 10 µM CM-H_2_DCFDA for 30 minutes at 37°C in the dark. Cells were analyzed by FACScalibur flow cytometry using excitation and emission filters set at 485 and 525 nm, respectively [38]. The data were analyzed using CellQuestPro software (BD).

### Statistical analysis

Paired Student’s *t* test or One-way analysis of variance (ANOVA) with Turkey’s *post-hoc* analysis was used to determine the statistically significant differences among all experimental results. The data were expressed as means ± SD of three independent experiments. Group means were compared to consider the statistically significant differences at *p*<0.05. All data were analyzed using the Graph Prism Software version 5.

## Results

### The effects of capsaicin on HepG2 cell viability

**Figure 1:**
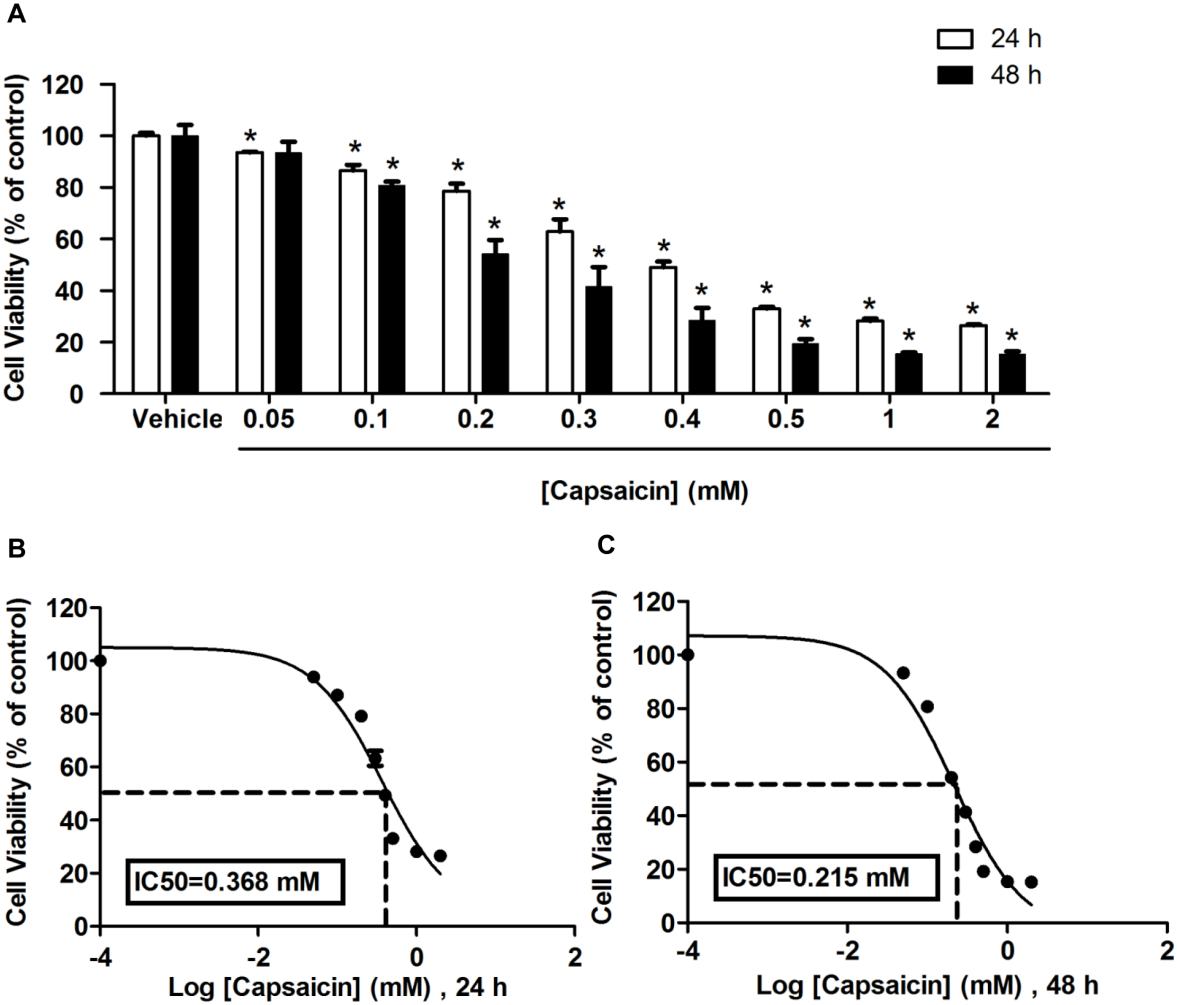
Effects of different concentrations of capsaicin on HepG2 cell viability. HepG2 cells were treated with capsaicin from 0.05 to 2 mM for 24 and 48 h. Data considered as the control were defined as cells treated with a medium or vehicles (0.1% DMSO) without capsaicin. (**A**) Effect of capsaicin on the proliferation of HepG2 cells was expressed as percentage of cell viability compared with 100% of the control. Effects of 24 (**B**) and 48 (**C**) h exposure to varying concentrations of capsaicin on cell proliferation are expressed as concentrations of capsaicin at which the viability of cells can be reduced to 50% (IC50). Data from at least three independent experiments performed in at least triplicates are presented as means ± SD, n = 9, *p<0.05.

To evaluate the anti-proliferative effects of capsaicin, a preliminary screening was performed first using the MTT assay. HepG2 cells were cultured and treated with capsaicin at 0 to 2 mM or with 0.1% DMSO vehicle for 24 and 48 h incubation periods. Capsaicin induced a reduction of the number of viable cells in a dose- and time-dependent manner, as shown in figure 1A. Incubation with capsaicin at the highest dose, 2 mM, for 24 h significantly decreased cell viability approximately up to 72% compared to the control group. Extending the incubation period to 48 h caused an augmentation of the anti-proliferation effect of capsaicin in HepG2 cells. The concentrations of capsaicin that were able to reduce the number of viable cells to 50% were 0.37 mM for 24 h (figure 1B) and 0.22 mM for 48 h (figure 1C).

### The effects of capsaicin on HepG2 cell apoptosis

**Figure 2:**
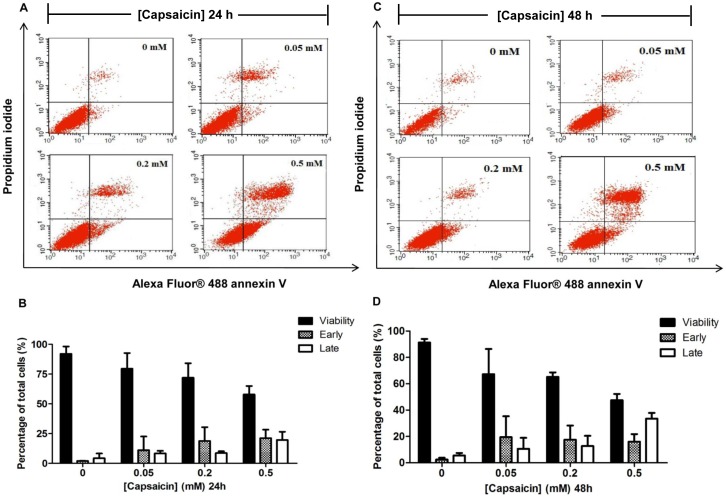
The effect of capsaicin on the rate of apoptosis on HepG2 cells. HepG2 cells were treated with 0.05, 0.2, and 0.5 mM of capsaicin for 24 h (**A, B**) and 48 h (**C, D**). The above panel shows a dual parametric dot plot (**A, C**) of cell population detection by flow cytometry depicting the distribution of viable, early, late apoptotic, and nec rotic cells evaluated by dual staining of PI fluorescence expressed on the y-axis versus Alex flour-Annexin V fluorescence expressed on the x-axis. The percentages (**B, D**) of cell populations relative to the whole cell populations (set as 100%) were expressed by bar charts showing the proportion of viable, early, and late apoptotic cells. The control was defined as cells treated with a medium or vehicles without capsaicin. Data from at least three independent experiments performed in at least triplicates are presented as means ± SD, n = 7.

To examine whether the cytotoxic effect of capsaicin on HepG2 cells was due to apoptotic death, HepG2 cells were exposed to varying concentrations of capsaicin and the rate of apoptosis was determined by flow cytometric analysis. In accordance with the cytotoxicity assay, we choose the concentration range that covered the logarithmic phase of the concentration-response (% cell viability) curve given by the MTT assay, which was 0.05, 0.2, and 0.5 mM for 24 and 48 h exposure of HepG2 cells to capsaicin. The specific characteristic of the early apoptosis event was assessed by changes of the plasma membrane symmetrical characteristic, the externalization of phosphatidylserine (PS). This could be observed as having a positive staining with Annexin V but negative staining with PI. However, the late apoptotic cells appeared to be stained with both Annexin V and PI. As shown in figure 2A to D, for the control group, cells that were exposed to medium or vehicles alone had negative staining of both Annexin V and PI. This indicated that they were the viable cells. The rate of early stage of apoptosis was increased after cells were treated with 0.5 mM capsaicin for 24 h (21.52%) and 48 h (16.06%) when compared with the control group (2.01–2.44%). The rate of late stage of apoptosis was obviously increased after cells were treated with 0.5 mM capsaicin for 24 h (17.79%) and 48 h (33.55%) when compared with the control group (4.28–5.53%). It was noticed that the percentage of viable cells decreased as the early and late apoptosis rates increased. At 0.5 mM capsaicin treatment for 24 h and 48 h caused the percentage of viable cells to decrease to 57.78% and 47.51%, respectively when compared to 92% of the control.

These results were also consistent with cell toxicity assayed by MTT and provided a strong suggestion that capsaicin caused cellular toxicity by induction cell apoptosis progression from early to late apoptosis. There was no effect of capsaicin on the rate of necrotic cells at all treatment doses and incubation time points.

### The effects of capsaicin on HepG2 cell apoptosis characterized by nuclear morphological changes

**Figure 3:**
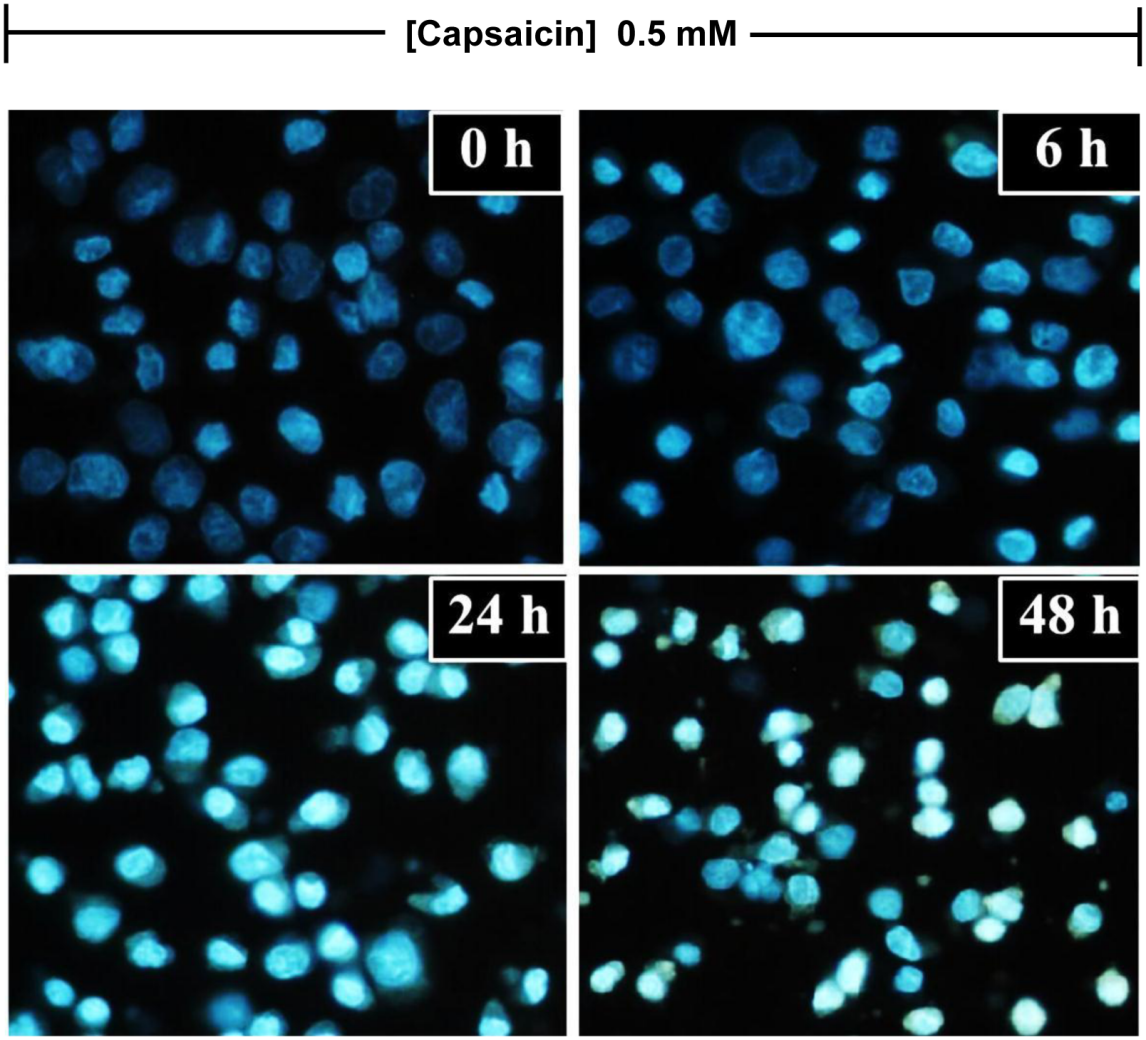
The effect of capsaicin on nuclear morphological alterations in HepG2 cells. HepG2 cells were incubated with capsaicin at concentrations of 0.05 mM for 0, 6, 24 and 48 h. DAPI dye was used to indicate the apoptotic morphological features, such as chromatin condensation, nuclear fragmentation, and membrane blebbing, and then visualized by fluorescence microscopy at 40x magnifications. The control was defined as cells treated with a medium or vehicles without capsaicin (Scale bar: 25 µm).

The effects of capsaicin on apoptosis were also characterized by hallmark apoptotic nuclear morphological characteristics, including chromatin condensation, nuclear fragmentation, and membrane blebbing. Attached and detached cells were collected and stained with DAPI. In the control group, cells had intact nuclei with a round shape and were uniformly stained (figure 3). HepG2 cells treated with capsaicin for 6 h showed remarkable nuclear changes which were a manifestation of apoptosis. These were pyknosis, which was the shrinkage and condensation of the nucleus, and karyorrhexis, which was the fragmentation of the nucleus. A prolonged incubation time period resulted in more prominent nuclear alterations.

### The effects of capsaicin on the cell cycle of HepG2 cells

**Figure 4:**
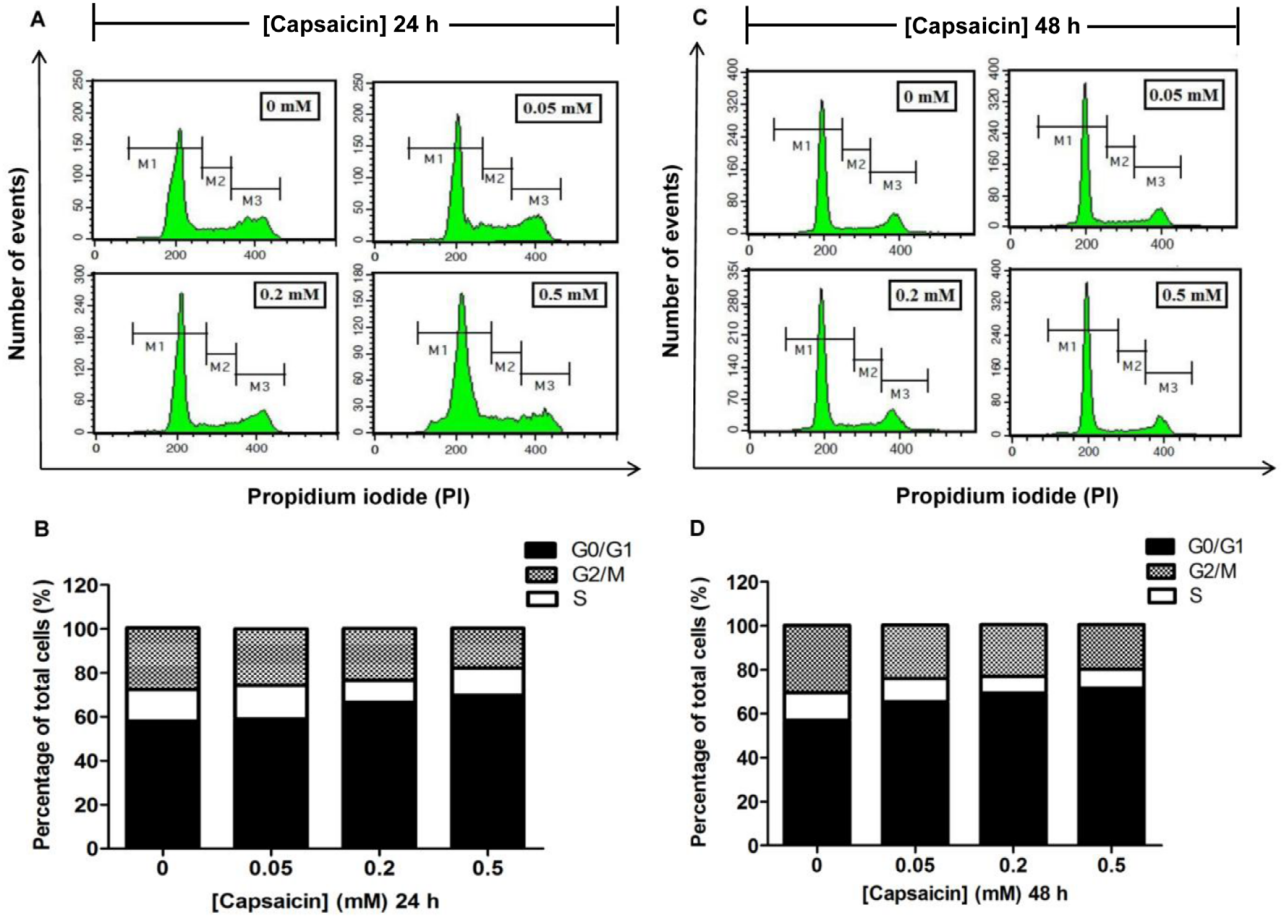
The effect of capsaicin on cell cycle distribution in HepG2 cells. HepG2 cells were incubated with 0.05, 0.2, and 0.5 mM of capsaicin for 24 h (**A, B**) and 48 h (**C, D**). The above panel (**A, C**) shows the histogram profile of cell cycle distribution in G0/G1, S, and G2/M phases detected by flow cytometry using PI staining. The bar chart (**B, D**) shows the percentage of cell populations in each phase of the cell cycle relative to the whole cell populations (set as 100%). The control was defined as cells treated with a medium or vehicles without capsaicin. Data are expressed as means ± SD from at least three separate experiments performed in triplicate, n = 3.

In this present study, a correlation of the apoptosis with the regulation of cell cycle progression was investigated. The proportion of cells in G0/G1, S, and G2/M phases after capsaicin treatment was determined using flow cytometry. At the indicated time points, attached and detached cells were collected, combined, and stained with PI. As shown in figure 4A to D, treatment with capsaicin induced an increase of cells in the G0/G1 phase of the cell cycle. The percentages of cells in the G0/G1 phase were 69.89% and 71.64% after treatment with 0.5 mM of capsaicin for 24 and 48 h, respectively, when compared with the control which had 57.99% of cells in the G0/G1 phase. Notably, the percentage of cells in the S and G2/M phases observed at 24 h and 48 h after capsaicin exposure decreased as the percentages of cells in the G0/G1 phase increased.

### The effects of capsaicin on the expression of FASN protein in HepG2 cells

**Figure 5:**
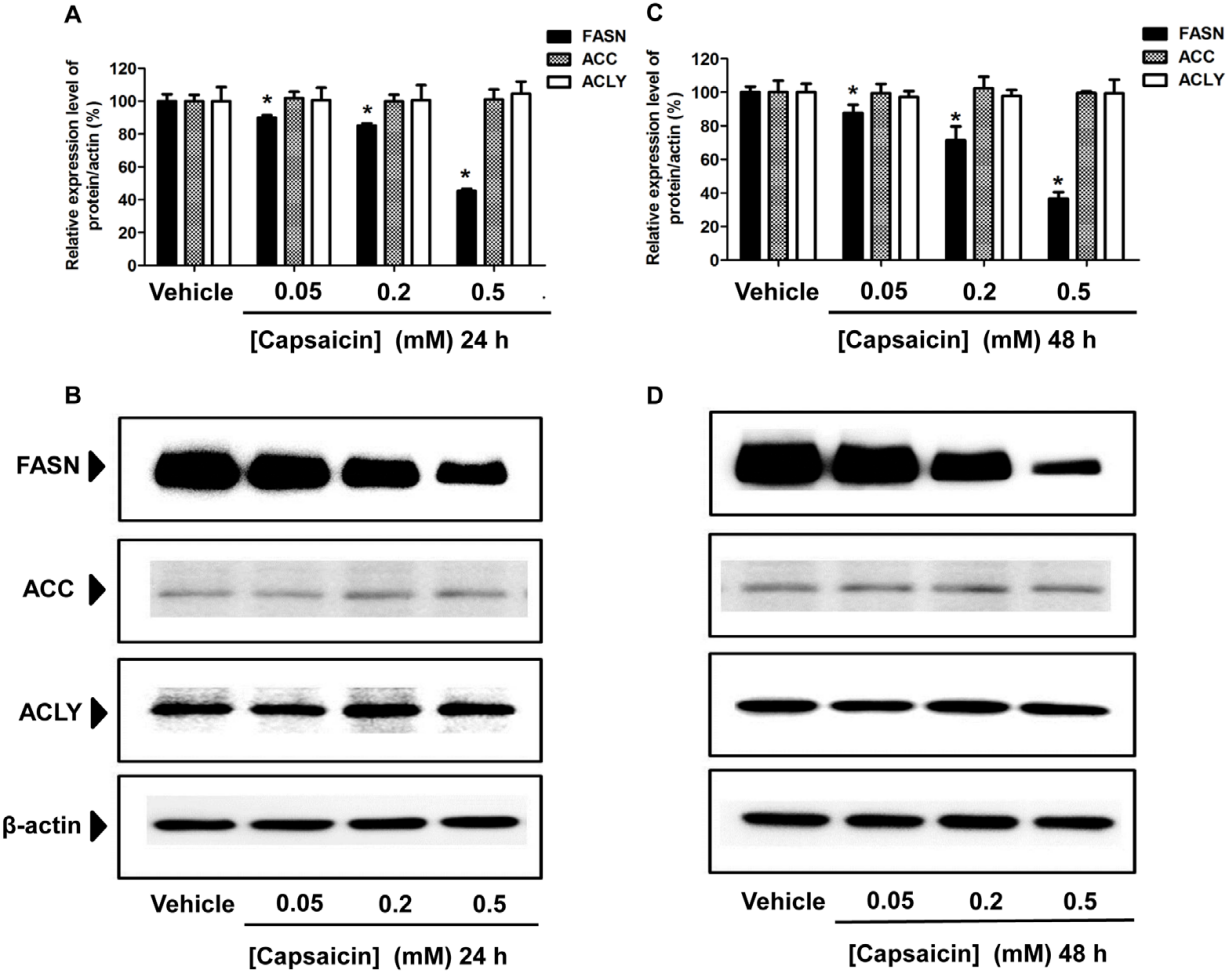
Effect of capsaicin on the expression of FASN, ACC, and ACLY protein levels in HepG2 cells. HepG2 cells were incubated with 0.05, 0.2, and 0.5 mM of capsaicin for 24 h (**A, B**) and 48 h (**C, D**), and then the cell lysates were subjected to the SDS-PAGE system. Protein expressions were detected by specific antibodies and. β-actin was used as an internal standard to confirm the integrity and equal protein loading. The bar graph (**A, C**) shows the quantification of band intensity as a ratio of the individual band intensity relative to the β-actin band intensity in the same blot (e.g. FASN/β-actin). The band (**B, D**) results shown are representative of those obtained from at least three independent experiments. Data are expressed as means ± SD from at least three separate experiments performed in triplicate, n = 5, *p<0.05.

To further investigate whether a decrease of the *de novo* synthesis of fatty acid by capsaicin was responsible for induction of apoptosis, the expression of target lipogenic enzyme FASN which catalyzes the synthesis of LCFAs in HepG2 cells was measured. It has been reported that the FASN enzyme is over-expressed in several malignant cells, while it is generally undetectable in normal, nonmalignant cells [30], [31], [32], [33]. Several studies have reported that pharmacologic inhibition of FASN expression and its enzymatic activity can induce apoptosis in cancer cell [39], [40], [41]. The present study determined the inhibitory effect of capsaicin on FASN expression and its enzymatic activity, which correlated with apoptosis in HepG2 cells. As shown in figure 5A and 5B, HepG2 cells treated with capsaicin at 0.5 mM for 24 h significantly caused approximately a 2-fold decrease of FASN protein expression compared with the control group which was exposed to a medium or vehicles without capsaicin. There was a small decrease of FASN expression observed at 0.2 mM capsaicin treatment for 24 h exposure. Prolonged incubation time to 48 h caused a much greater decrease of FASN protein expression levels, more than 3-fold compared to the control group (figure 5C and 5D). However, no significant effect of capsaicin exposure for 24 h and 48 h at a concentration of 0.05 mM was observed on the suppression of FASN expression. Taken together, these results suggest that the inhibitory effect of capsaicin on FASN protein expression occurred in a dose- and time-dependent manner. Capsaicin-induced apoptosis in HepG2 is involved in a reduction of FASN protein expression.

This study also determined whether an alteration of the expression of FASN protein level was accompanied by variation of other lipogenic enzymes responsible for the *de novo* synthesis of fatty acid, such as acetyl-CoA-carboxylase (ACC) and ATP-citrate lase (ACLY) after capsaicin treatment. Considerable amounts of ACC and ACLY protein expression, albeit to a lesser extent than FASN expression, were found in HepG2 cells. The expression of ACC and ACLY proteins was approximately 5-fold lower than that of FASN proteins. Interestingly, capsaicin did not alter the expression of ACC and ACLY protein levels in HepG2 cells.

### The effects of capsaicin on FASN activity in HepG2 cells using intracellular fatty acid and triglyceride quantification assays

**Figure 6:**
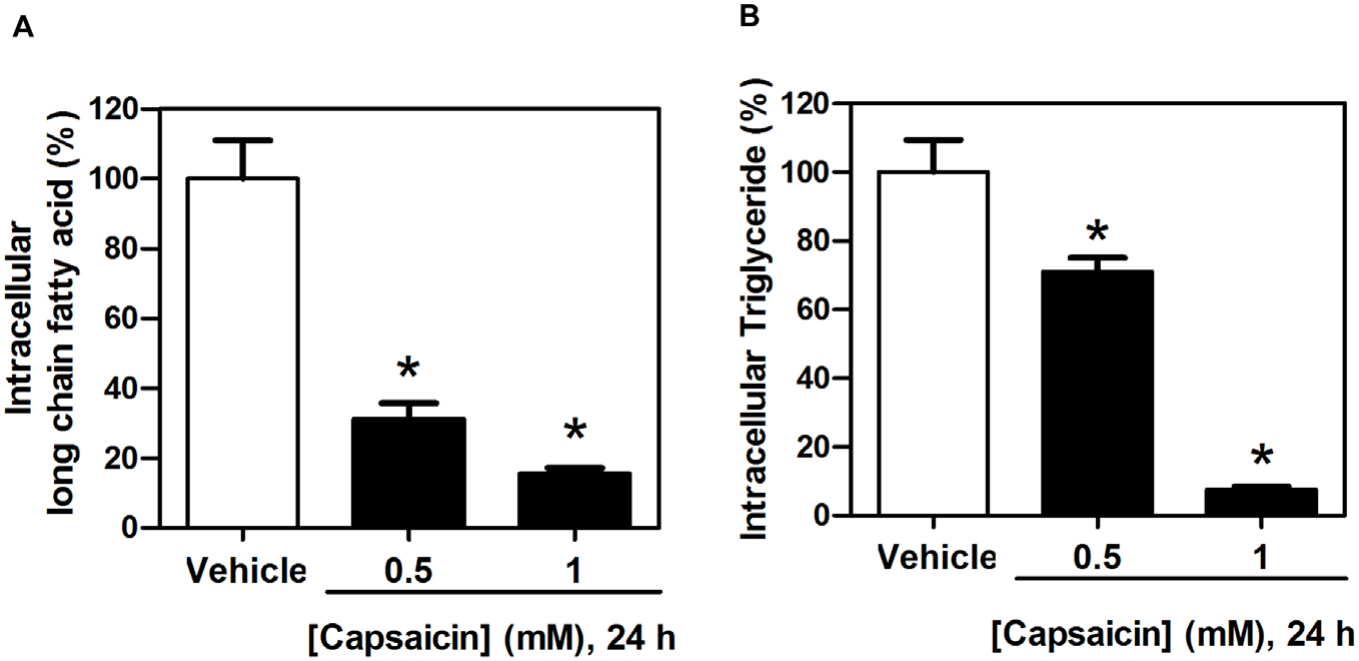
Effects of capsaicin on fatty acid synthesis in HepG2 cells. Intracellular long chain fatty acid (**A**) and intracellular triglyceride (**B**) synthesis were determined by using the free fatty acid and triglyceride quantification kits. HepG2 cells were incubated with 0.5 and 1.0 mM of capsaicin for 24 h. The control was defined as cells treated with a medium or vehicles without capsaicin. Data from at least three independent experiments performed in triplicate are represented as percentage of individual value in nmol/L compared to 100% of control values and are shown as means ± SD, n = 3, *p<0.05.

To assess additional supporting evidence for the potential effects of capsaicin that suppressed the expression of FASN protein and *de novo* fatty acid synthesis in HepG2 cells, FASN activity was analyzed by measuring the levels of intracellular long chain free fatty acids and triglycerides. In agreement with previous reports, the down-regulated FASN protein expression was accompanied by decreased enzymatic FASN activity in intracellular fatty acid and triglyceride synthesis [30]. Treatment of HepG2 cells with capsaicin at 0.5 mM for 24 h reduced intracellular long chain fatty acid levels more than 60% compared to 100% of the control (figure 6A). Capsaicin at the concentration of 0.5 mM for 24 h also caused a decrease in triglyceride levels by 30% as shown in figure 6B. In addition, the levels of fatty acid and triglyceride were reduced more as the capsaicin concentration increased. Our results demonstrated that capsaicin-induced down-regulation of the expression of FASN protein levels in HepG2 cells was accompanied with a marked suppression of the *de novo* fatty acid synthesis pathway.

### Capsaicin induced mitochondrial dependent apoptosis in HepG2 cells

**Figure 7:**
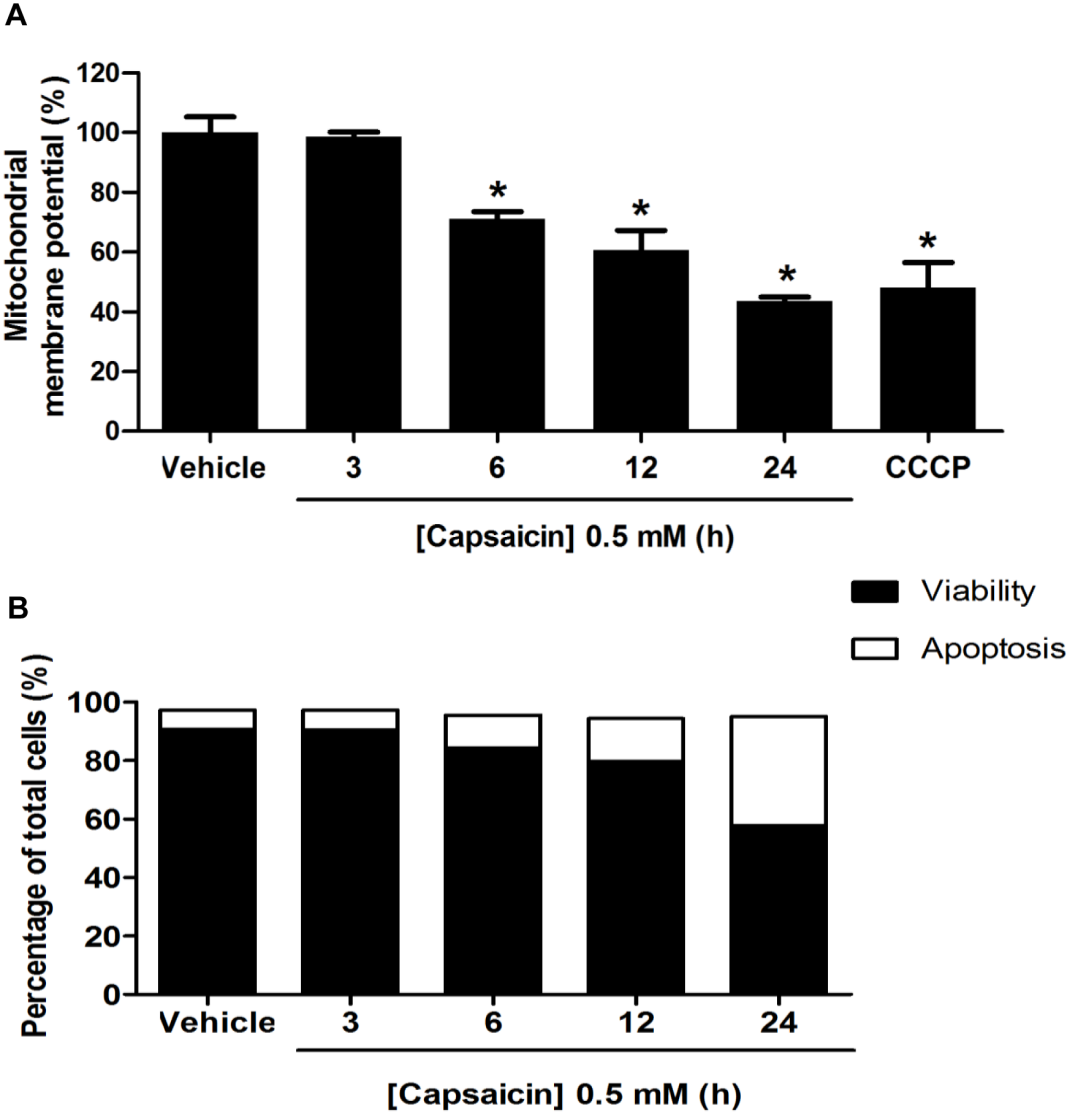
Effect of capsaicin on induction of mitochondrial dependent apoptosis in HepG2 cells. HepG2 cells were incubated with 0.5 mM capsaicin for 3 to 24 h. (**A**) The ΔΨm was examined by 2 µM JC-1 dye and detected by flow cytometry. The control was defined as cells treated with a medium or 0.1% DMSO vehicle without capsaicin. Histograms show the percentage of an energized and high polarization mitochondrial state as calculated by a ratio of red and green fluorescence intensity compared to the control. The decrease in percentage of ΔΨm represented a decrease in the red to green fluorescence intensity ratio. CCCP was used as a positive control to induce disruption of ΔΨm. (**B**) The effect of capsaicin on the rate of apoptosis on HepG2 cells following 0.5 mM capsaicin treatment for 3 to 24 h. The percentages of cell populations were expressed as bar charts showing the proportion of viable and apoptotic cells detected by flow cytometry using dual staining of PI and Alex flour-Annexin V fluorescence. Data from at least three independent experiments performed in at least triplicates are presented as means ± SD, n = 3.

To further evaluate whether the apoptosis induction by capsaicin in HepG2 cells is initiated via the disruption of ΔΨm, which is now accepted as one of the early apoptotic features [42], [43]. The ΔΨm was assessed by flow cytometry using an indicator JC-1 fluorescence dye. With a high ΔΨm, JC-1 appears in aggregated form that emits at 590 nm (red fluorescence), whereas at a low ΔΨm, it becomes a monomeric form and emits at 530 nm (green fluorescence). The level of red to green fluorescence emission ratio indicates an energized and polarization mitochondrial condition. The decrease in the red to green fluorescence intensity ratio indicates a decrease in ΔΨm. As shown in figure 7A, the death of HepG2 cells by apoptosis was associated with a decrease of ΔΨm. The ΔΨm began to decrease approximately 29% after HepG2 cells were treated with 0.5 mM of capsaicin for 6 h compared with the control group. Cells treated with capsaicin for 3 h did not show any sign of a decrease of ΔΨm. Prolonged incubation time caused more prominent damage of ΔΨm. Consistent with the loss of ΔΨm shown in figure 7B, an increased rate of apoptosis by 11.30% was initially detectable at least 6 h after capsaicin exposure.

### The effects of C75 on FASN expression, fatty acid synthesis, and apoptosis in HepG2 cells

**Figure 8:**
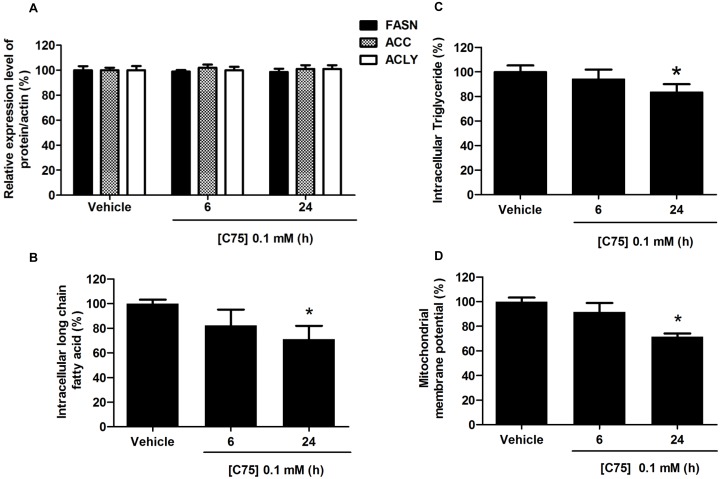
Effects of C75 on FASN protein expression, free fatty acid levels, triglyceride levels, and disruption of ΔΨm in HepG2 cells. HepG2 cells were treated with C75 at 0.1 mM concentration for 6 h and 24 h. The control was defined as cells treated with a medium or vehicles without C75. (**A**) For the effect of capsaicin on the expression of FASN protein level in HepG2 cells, β-actin was used as the internal standard. The bar graph shows the quantification of band intensity as a ratio of FASN protein band intensity relative to the β-actin band intensity in the same blot (e.g. FASN/β-actin). For the effect of capsaicin on intracellular long chain fatty acid (**B**) and triglyceride synthesis (**C**) were determined by using the free fatty acid and triglyceride quantification kits, data are represented as percentage of the individual value in nmol/L compared to 100% of control values. (**D**) The bar chart shows the percentage of disruption of ΔΨm compared to the control (set as 100%). The change in ΔΨm was determined by staining cells with JC-1 fluorescence dye and detected by flow cytometry. Data from at least three independent experiments performed in triplicate are shown as means ± SD, n = 3, *p<0.05.

In this present study, we found that C75, the pharmacologic inhibitor of FASN [22], at 0.1 mM significantly decreased the free fatty acid and triglyceride levels by 29% and 12%, respectively, which were initially visible at 24 h after exposure, as shown in figure 8B and C, without any change in the level of FASN protein expression (figure 8A). We observed a similar decrease of ΔΨm by 23% following treatment with 0.1 mM of C75 for 24 h (figure 8D). Our results are in agreement with previous studies that C75 induces apoptosis via a direct suppression of FASN activity and fatty acid synthesis, without changes in the abundance of FASN protein expression [34], [39].

### Induction of apoptosis in HepG2 cells was triggered by the inhibition of FASN expression and fatty acid synthesis

**Figure 9:**
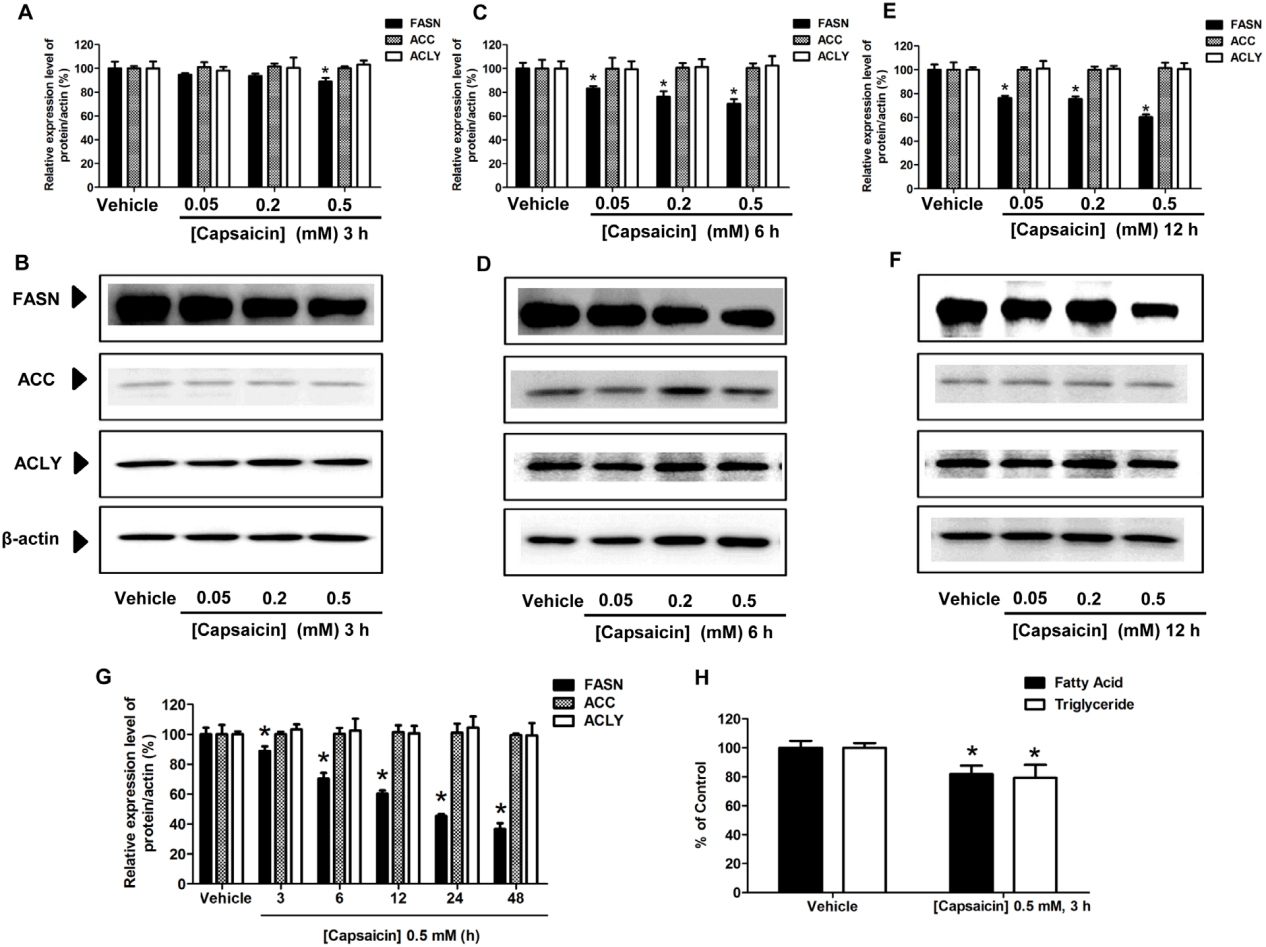
Effect of capsaicin on the expression of FASN, ACC, and ACLY protein levels in HepG2 cells. HepG2 cells were incubated with 0.05, 0.2, and 0.5 mM of capsaicin for 3 h (**A, B**), 6 h (**C, D**), and 12 h (**E, F**). (**G**) Histograms show the time-course effect of 0.5 mM capsaicin on protein expression in HepG2 cells for 3 to 48 h. Equal amounts of total protein were subjected to immunoblotting analysis with specific antibodies. β-actin was used as the internal standard to confirm the integrity and equal protein loading. Immunoreactive bands shown are representative of those obtained from at least three independent experiments. The bar graph shows the quantification of band intensity as a ratio of the individual band intensity relative to the β-actin band intensity in the same blot (e.g. FASN/β-actin). (**H**) Intracellular long chain fatty acid and triglyceride synthesis were determined by using the free fatty acid and triglyceride quantification kits following 0.5 mM capsaicin treatment for 3 h. The control was defined as cells treated with a medium or vehicles without capsaicin. Data are expressed as means ± SD from at least three separate experiments performed in triplicate, n = 3, *p<0.05.

The present study aimed to investigate whether capsaicin-suppressed fatty acid synthesis was an upstream event of apoptosis induction in HepG2 cells. As shown in figure 9A to G, FASN protein expression initially decreased to 88.81% from 100% of the control after 3 h of exposure to a 0.5 mM of capsaicin and more prominently decreased after a prolonged incubation period. After 3 h incubation with 0.5 mM of capsaicin, free fatty acid and triglyceride levels were decreased by 20% (figure 9H). Taken together, these results support the notion that inhibition of FASN protein expression and fatty acid synthesis precedes the onset of ΔΨm and apoptosis which showed evidence at least 6 h after capsaicin exposure (figure 7A and 7B). It verifies that capsaicin down-regulates FASN protein expression, which in turn triggers the mitochondrial-dependent apoptosis pathway in HepG2 cells.

### The inhibitory effect of capsaicin on FASN protein expression and activity inducing disruption of ΔΨm and apoptosis was mediated by ROS production

**Figure 10:**
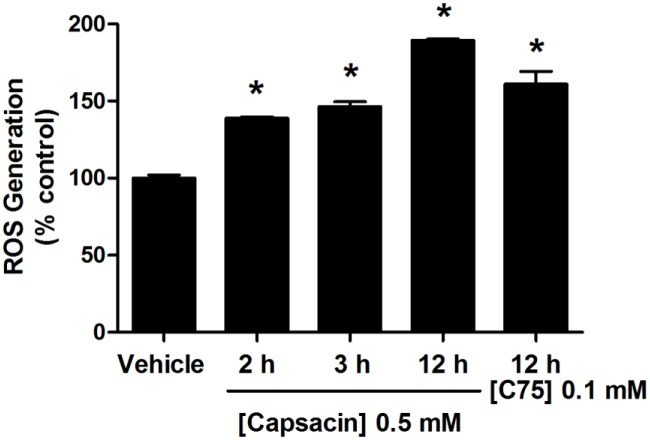
Effect of capsaicin on ROS generation in HepG2 cells. HepG2 cells were treated with 0.5 mM of capsaicin for 2, 3, and 12 h. The control was defined as cells treated with a medium or 0.1% DMSO vehicle without capsaicin. ROS production was measured by CM-H_2_DCFDA fluorescent dye and detected by flow cytometry. Representative histogram plots using flow cytometric analysis indicate percentage of ROS generation compared to the control group (100%). ROS production following 0.1 mM C75 treatment for 12 h was measured. Data are expressed as means ± SD from at least three separate experiments performed in triplicate, n = 3, *p<0.05.

This study investigated whether ROS generation was a preceding event of capsaicin induced inhibition of fatty acid synthesis and apoptosis in HepG2 cells. Figure 10 shows that the percentage of intracellular ROS generation increased from 100% of the control to approximately 139% after 2 h of 0.5 mM capsaicin treatment, which preceded the onset of a down-regulation of FASN protein expression (figure 9) and damage of ΔΨm (figure 7). Prolonged treatment with capsaicin to 3 h and 12 h exhibited a prominent ROS generation to approximately 146% and 189%, respectively. Taken together, these findings suggest that capsaicin increases ROS production, which in turn suppresses FASN expression and the *de novo* fatty acid synthesis pathway, inducing mitochondrial dependent apoptosis in HepG2 cells. We also observed an increase of ROS generation to approximately 150% following 0.1 mM C75 treatment for 12 h, consistent with a previous report in human embryonic kidney (HEK293T) cells [44].

### An accumulation of malonyl-CoA following an inhibition of fatty acid synthesis by capsaicin was a major cause of apoptosis induction in HepG2 cells

**Figure 11:**
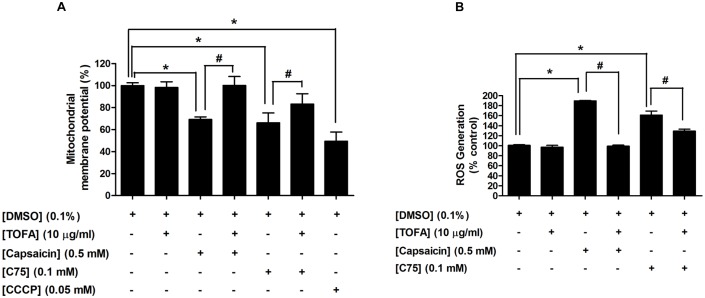
An accumulation of Malonyl-CoA plays a vital role in capsaicin-induced apoptosis in HepG2 cells. HepG2 cells were treated with 0.5 mM capsaicin or 0.1 mM C75 or 10 µg/ml TOFA for 12 h. Co-incubation of capsaicin or C75 with TOFA was performed by pre-incubation TOFA 1 h before treatment cells with capsaicin or C75. The control was defined as cells treated with a medium or 0.1%DMSO vehicle alone. (**A**) The ΔΨm was examined by 2 µM JC-1 dye and detected by flow cytometry. Histograms show the percentage of an energized and high polarization mitochondrial state as calculated by a ratio of red and green fluorescence intensity compared to the control. The decrease in percentage of ΔΨm represented a decrease in the red to green fluorescence intensity ratio. CCCP at 0.05 mM was used as a positive control to induce disruption of ΔΨm. (**B**) ROS production was measured by CM-H_2_DCFDA fluorescent dye and detected by flow cytometry. Histogram plots using flow cytometric analysis indicates percentage of ROS generation compared to the control group (100%). Data are expressed as means ± SD from at least three separate experiments performed in triplicate, n = 3, *p<0.05.

Several previous studies have reported that a reduction of end product fatty acids *per se* or an accumulation of malonyl-CoA, as a result from FASN inhibition, appears to be one of the major causes of apoptosis induction [19], [45]. This experiment was designed to investigate whether the apoptosis induction by FASN inhibition after capsaicin treatment is mediated by a reduction of end product fatty acids *per se* or an accumulation of malonyl-CoA. Following treated cells with a competitive inhibitor of ACC, 5-(tetradecyloxy)-2-furoic acid (TOFA) at 10 µg/ml for 12 h, an impairment of ΔΨm was not observed, while 0.5 mM capsaicin and 0.1 mM C75 treatment for 12 h showed reduction of ΔΨm to approximately 69.2% and 66.17%, respectively compared to 100% of the control, as shown in figure 11A. It is therefore, possible that a reduction of fatty acid *per se* is not the mediator of apoptosis induction following inhibition of FASN expression and the fatty acid synthesis pathway. To evaluate the possibility that accumulation of malonyl-CoA is a major mediator to trigger disruption of ΔΨm after inhibition of fatty acid synthesis, this study performed by exposed cells to TOFA for 1 h prior to 0.5 mM capsaicin and 0.1 mM C75 treatment for 12 h. Application of TOFA 1 h before treatment with capsaicin to HepG2 cells reduced the cytotoxic effect of capsaicin and reversed the damage of ΔΨm back to the control state. Thus, this result demonstrates that an accumulation of malonyl-CoA, following FASN inhibition by capsaicin appears to play a more important role than the depletion of end product fatty acids on induction of damage of ΔΨm and apoptosis in HepG2 cells, consistent with a previous report [46]. We observed a similar partial rescue potency of TOFA on C75, which disrupted ΔΨm for 12 h of treatment. In this study, CCCP was used as a positive control to induce depolarization of ΔΨm.

Figure 11B shows that administration of TOFA 1 h prior to treatment of HepG2 cells with 0.5 mM capsaicin for 12 h completely blocked the generation of ROS from approximately 260% back to 100% of the control. Deprivation of fatty acid alone following TOFA administration failed to increase ROS production. Thus, we suggest that accumulation of malonyl-CoA following FASN inhibition by capsaicin might be able to cause ROS generation, correlating with mitochondrial impairment and apoptosis induction in HepG2 cells.

### Selective induction of apoptosis by capsaicin-mediated FASN inhibition was found in HepG2 cells but not in normal human hepatocytes

**Figure 12:**
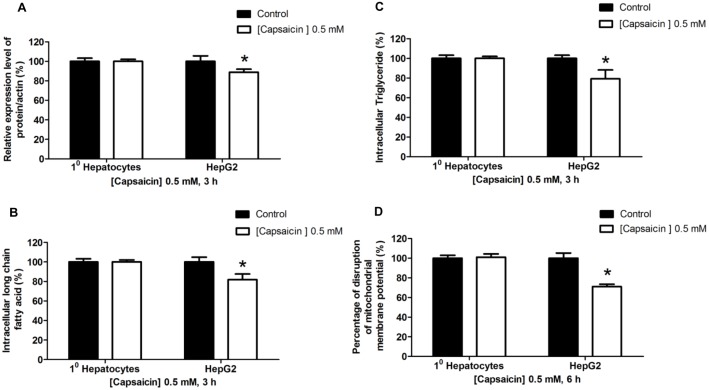
Effects of capsaicin on normal human hepatocytes compared with HepG2 cells. Cells were treated with 0.5 mM concentration of capsaicin for 3 h or 6 has indicated. The control was defined as cells treated with a medium or vehicles without capsaicin. (**A**) The bar graph shows the quantification of band intensity as a ratio of FASN protein band intensity relative to the β-actin band intensity in the same blot (e.g. FASN/β-actin). (**B**) For the effect of capsaicin on intracellular long chain fatty acid and (**C**) triglyceride synthesis determined by using the free fatty acid and triglyceride quantification kit, data are represented as percentage of the individual value in nmol/µL compared to 100% of control values. (**D**) The bar chart shows the percentage of disruption of mitochondrial membrane potential relative to the control (set as 100%) after 6 h of capsaicin treatment by JC-1 fluorescence dye staining and detected by flow cytometry. Data from at least three independent experiments performed in triplicate are shown as means ± SD, n = 3, *p<0.05.

This study evaluated the selective impact of capsaicin on apoptotic induction mediated by FASN inhibition on HepG2 cells but not on normal human hepatocytes. We used normal human hepatocytes to represent control cells which have low FASN expression and enzymatic activities. The present finding is consistent with previous studies that showed that normal hepatocytes were characterized by a 5-fold lower FASN protein expression (data not shown), lower fatty acid, and triglyceride synthesis than HepG2 cells, as shown in figure 12A to C. Previous studies have shown that FASN expression and its activity in normal cells are many times lower than that in cancer cells [30]. Hepatocytes treated with 0.5 mM of capsaicin for 3 h had a comparable level of FASN protein expression and enzymatic activities to the control group. Furthermore, hepatocytes treated with 0.5 mM capsaicin for 6 h did not show any changes of mitochondrial integrity as shown in figure 12D. These findings clearly suggest that capsaicin inducing FASN inhibition and enzymatic activities leading to induction apoptosis is selective in HepG2 cells but not in normal human hepatocytes. Thus, it affirms that FASN would be a potential target for anti-cancer therapy of capsaicin.

## Discussion

Capsaicin has been found to possess anti-cancer properties via the stimulation of apoptosis in various human cancer cell lines [13], [47]. This study reports that treatment of HepG2 cells with capsaicin results in a decrease in cell proliferation, an enhancement of cell cycle arrest, and an induction of cell apoptosis. A correlation between apoptosis induction and upstream FASN expression inhibition was observed, raising the possibility that inhibition of FASN by capsaicin triggers apoptosis in HepG2 cells. There have been no studies evaluating the impact of FASN inhibition following capsaicin exposure in induction of apoptosis in HepG2 cells. The present study found that capsaicin-induced apoptosis was restricted to cancer cells while normal hepatocyte cells were resistance to capsaicin. Furthermore, apoptosis induction by capsaicin was observed to be related to the generation of ROS, which is considered as a mediator to suppress FASN expression leading to disruption of ΔΨm.

This study provides evidence that FASN expression is several folds up-regulated in HepG2 cells and exhibits responsiveness to capsaicin compared to normal human hepatocytes. Consistent with other reports, FASN expression is markedly detected in most epithelial cancer cells, whereas epithelial cells of normal tissues have lower expression. The liver is one of the lipogenic organs that show more expression levels of FASN than other normal tissues [45], [48], [49]. Such an over-expression of FASN observed in cancer cells is, therefore, considered the important biological role of this enzyme in tumorigenesis [22], [28]. The present study also reports that the expression of acetyl-CoA carboxylase (ACC) and ATP-citrate lyase enzyme (ACLY) protein levels are higher in high FASN expressing HepG2 cells than in normal hepatocytes. Indeed, this study showed that capsaicin is unable to alter ACC and ACLY protein expression levels, suggesting that ACC and ACLY are not the specific targets of the cytotoxic effect of capsaicin in HepG2 cells. Consistent with this concept, the present study affirms that capsaicin has an inhibitory effect on FASN activity similar to C75, the well-known chemical inhibitor of FASN activity [50], blocking fatty acid synthesis, which in turn induces apoptosis in HepG2 cells. Its effect is selectively observed in HepG2 cells but not in normal liver cells. These results suggest that capsaicin is, therefore, a selective inhibitor of *de novo* fatty acid synthesis through inhibition of FASN expression and activity. The overexpression of FASN in cancer cells has been reported to be regulated by a single oncogene, *Akt*, and HER-2 pathways through the hypoxic-inducible factor-1α (HIF-1α) in breast cancer cells [51]. In addition, the regulation of protein stability and degradation by a signaling protein β-catenin in mantle cell lymphoma (MCL) [29] as well as post-transcriptional regulation by ubiquitin-specific-protease 2a (USP2a) and isopeptidase proteins breast cancer cells [52] also up-regulate the expression of FASN. β-catenin can modulate USP2a that results in promoting FASN stabilization leading to an increase the expression of the FASN protein [29]. Thus, the high level of FASN expression in HepG2 cells is essential for cancer cell proliferation and responsiveness to capsaicin or other FASN inhibitors. In contrast, normal cells having much lower FASN expression exhibit resistance to capsaicin.

However, there is still the question of which mechanism mediates the inhibitory effect of capsaicin on suppression of FASN expression and its activity on fatty acid synthesis in cancer cells. Herein, following capsaicin exposure, we observed an increase of ROS generation preceding an inhibition of FASN expression, leading to dissipation of ΔΨm and apoptosis. We suggest that ROS is an important cellular mediator that triggers the intrinsic- and mitochondrial-dependent apoptotic pathway through depletion of fatty acid synthesis after administration of capsaicin in HepG2 cells. Our suggestion is in agreement with other studies reporting that capsaicin coupled with the TRPV1 receptor and exerted apoptosis is mediated by an increase of ROS production in human cancer cells, such as nasopharyngeal carcinoma [13], colon cancer cells [53], and bladder cancer T24 cells [54]. Besides ROS-mediated apoptosis, several studies have reported that the effect of capsaicin to induce apoptosis is mediated by an increase of intracellular calcium following activation of the TRPV1 receptor in glioma cells [9] and human urothelial cancer (UC) cells [55]. Elevation of intracellular calcium and ROS levels have also been reported to be mediators of the FASN inhibitor on inhibition of FASN activity and induction of apoptosis in mouse metastatic melanoma cell line B16-F10 [56]. However, the mechanisms by which capsaicin triggers ROS generation have not been demonstrated in the present study. Recent study in pancreatic cancer cells demonstrates that inhibition of mitochondrial electron transport chain (ETC) complexes-I and III activities is the source of ROS generation following capsaicin exposure. Capsaicin also induces a depletion of antioxidants levels and activities which contribute to the accumulation of ROS [6].

The exact mechanism for excessive ROS generation retarding FASN expression and its fatty acid synthesis activity, inducing apoptosis remains unclear. Increase production of ROS after orlistat administration has been implicated in the elevation of IFN-γ, which manifests induction of apoptosis through inhibition of FASN expression in Dalton's lymphoma-transplanted BALB/c mice [57]. Indeed, over production of ROS in cancer cells causes impairment of cellular redox homeostasis inducing oxidative stress that consequently can make cancer cells more susceptible to oxidative stress than normal cells, which effectively balance the redox system [3], [6]. Recent study has reported that ROS generation causes oxidation of mitochondria cardiolipin, which promotes liberation of cytochrome c into cytosol for the disruption of mitochondria, leading to apoptosis in pancreatic cancer cells [6]. Elevation of ROS following capsaicin treatment exerts apoptotic induction via oxidative stress to mitochondrial megachannel pores which leads to damage of ΔΨm and apoptosis in colon cancer cells [58]. ROS-induced oxidative stress triggers the release of lysosomal protease cathepsin D into the cytosol which leads to induction of cytochrome c released from mitochondria and activation of apoptotic cascades in prostate cancer cells [59]. We also postulated the possibility that the generation of ROS-induced oxidative stress may modulate the activity of signaling machinery targets that function to regulate FASN protein expression. This hypothesis is supported by previous studies reporting that decreased FASN activity by C75 and (−)-epigallocatechin-3-gallate (EGCG) can induce apoptosis through the downregulation of phosphorylated and total protein levels of the epidermal growth factor receptor (EGFR) and its downstream phosphorylated form of cellular signaling pathways, including extra cellular-signal-regulated kinase (p-ERK1/2), p-AKT, and p-mTOR in A549 lung carcinoma cells [34]. ROS can be generated from mitochondria and cytosolic sources following EGCG exposure [60], [61]. Furthermore, additional studies suggest that EGCG can also decrease the phosphorylated forms of ERK and mitogen-activated protein (MEK1) through its oxidative stress inhibition of the association between Raf-1 and MEK1/ERK kinase1, leading to suppression of proliferation of the HT29 human colon cancer cell line [62].

FASN expression has been reported to be regulated by the human epidermal growth factor receptor HER2 oncogene (erbB2) through phosphatidylinositol 3-kinase (PI3-K)/Akt in breast cancers [63] as well as by mTOR signaling pathways in colorectal cancer cells [64]. Inhibition of HER2 expression can suppress Akt, mTOR, and JNK activity that leads to suppression of FASN expression and induction of apoptosis. Indeed, recent studies have obtained similar results, showing that the transcription of FASN is enhanced by activation of phosphatidylinositol 3-kinase (PI3-K) and mitogen-activated protein (MAP) kinase pathways through the key transcriptional factor sterol regulatory element-binding protein 1 (SREBP-1) and SREBP-2 in several cancer cells [18], [65] as well as in HepG2 cells. Taken together, we propose that the apoptotic activity of capsaicin is mediated by a key mediator ROS that can be able to suppress FASN expression through dysregulation of several intracellular signaling pathways.

The present study noted that the degree of deprivation of FASN expression and its activity on fatty acid synthesis is similar to the degree of mitochondrial damage and apoptosis. Our results confirm that the lipogenic enzyme FASN is the major attributory cause of induction of apoptosis in HepG2 cells following capsaicin administration. However, the mechanism explaining how deprivation of FASN expression and fatty acid synthesis contribute to apoptosis in cancer cells is still not clear. We postulate that inhibition of FASN impairs the production of fatty acid levels responsible for membrane formation, intracellular signaling pathways, and protein modification at post-translational process as well as storage of energy in cancer cell proliferation process [29]. More than 90% of the 16-carbon long-chain saturated fatty acid product, palmitate is primarily produced by the activity of FASN whereas FASN contributes to only 25% of cholesterol and cholesterol ester synthesis [49], [66]. However, suppression of FASN by RNAi in the human prostatic cancer cell line LNCaP reduces fatty acid synthesis without an alteration of cholesterol synthesis [30]. Additionally, it has been reported that the mitochondrial dysfunction following FASN inhibition can further induce apoptotic cell death through stimulation of ROS generation and disruption of respiratory chain complexes I and II by caspase-3 activation [67]. Inhibition of FASN also enhances accumulation of cofactor nicotinamide adenine dinucleotide phosphate (NADPH) that, therefore, leads it to be transformed into ROS in cancer cells [68].

As a result of over-expression of FASN protein and its activity, cancer cells exhibit the high requirements of fatty acid products for the synthesis of membrane phospholipid. The newly synthesized endogenous fatty acids also play a crucial role in supplying diacylglycerol which is the precursor for phospholipid. The major form of phospholipid in proliferating cells is phosphatidylcholine [49], [66]. However, when the rate of phosphatidylcholine synthesis decreases, diacylglycerol is able to convert into triglyceride pools. In addition to major membrane phospholipid content, phosphatidylcholine also functions as precursor for sphingomyelin and phosphatidylethanolamine synthesis. They are the other two major membrane phospholipids. Maximum increase in their biosynthesis has been reported during the S phase of cell division [69]. The present study suggests that a reduction of *de novo* synthesis of fatty acid leads to a decrease of diacylglycerol synthesis and consequently reduces membrane phospholipid synthesis. As a consequence of membrane phospholipid depletion, the cell undergoes cell cycle arrest and apoptosis [66]. Indeed, reduction of membrane biosynthesis following administration of orlistat exerts a decline expression of membrane associated pH regulators: MCT-1 and VH^+^ ATPase, which consequently induces alkalinization of tumor microenvironment and apoptosis in Dalton's lymphoma-transplanted mice [57]. Inhibition of FASN also elevates the level of polyunsaturated fatty acids and decreases the level of monounsaturated fatty acids which consequently leads to apoptosis via activation of lipid peroxidation and oxidative stress [70]. The levels of phospholipids were not examined in this study; however, a reduction of long-chain free fatty acid level would indirectly indicate the concomitant decrease of a precursor for phospholipid synthesis.

This study found that treatment of cancer cells with a competitive inhibitor of ACC, 5-(tetradecyloxy)-2-furoic acid (TOFA) inhibits fatty acid synthesis without toxicity to this cancer cell. Thus, a reduction of end product fatty acids *per se* seems not to be sufficient to account for the apoptosis induced by FASN inhibition in HepG2 cells. Besides a deficiency of synthesized fatty acids, inhibition of FASN also results in an accumulation of fatty acid intermediate malonyl-CoA [19], [22], [29]. An inhibition of FASN can contribute to an accumulation of malonyl-CoA by reducing the utilization of the malonyl-CoA precursor to synthesize fatty acids. A reduction of FASN, in addition to decreasing the levels of free fatty acid palmitate, contributes in part to a diminution of the inhibitory effect in ACC activity, allowing the activated ACC to augment the synthesis of malonyl-CoA [22], [45]. Inhibition of malonyl-CoA synthesis by TOFA has been shown to play a protective role against apoptosis induced by synthetic FASN inhibitors [22], [66]. Taken together, we suggest that an accumulation of a fatty acid pathway intermediate, malonyl-CoA, following FASN inhibition appears to be one of the major causes of apoptosis, in addition to a depletion of end product fatty acids in HepG2 cells. This finding has been affirmed by other studies where malonyl-CoA was characterized as a pro-apoptotic factor [66].

The mechanism of the cytotoxicity of high malonyl-CoA levels appears to be involved with an inhibition of the β-oxidation of the fatty acid pathway by suppressing carnitine palmitoyltransferase-I (CPT-1) activity that is localized at the outer mitochondrial membrane [22], [56]. CPT I which is a component of the mitochondrial CPT enzyme complex facilitates the transport of palmitoyl-CoA into the mitochondrial matrix for β-oxidation and for sphingolipid synthesis [71]. Elevation of malonyl-CoA levels by FASN inhibitors is accompanied by an accumulation of sphingolipid ceramide. The accumulation of ceramide decreases the removal of fatty acyl-CoA, a sphingolipid precursor, from cytoplasm via β-oxidation, leading to enhancing apoptosis [72]. Moreover, ROS generation after capsaicin treatment may enhance ceramide accumulation resulting from the hydrolysis of membrane sphingomyelin by the enzyme sphingomyelinase [15]. Ceramide, one of the two main sphingolipids, plays a regulatory role in cellular processes, including cell arrest, cell death, and apoptosis mediated by death inducers [22], [73]. Ceramide may facilitate apoptosis by activating pro-apoptotic genes, such as *BINP3*, *a tumor necrosis factor (TNF-related apoptosis-inducing ligand (TRAIL)*, and *death-associated protein kinase 2 (DAPK2)*. This consequently induces mitochondrial dysfunction and apoptosis [19]. In addition, ceramide activates a downstream protease prICE that cleaves an enzyme poly (ADP-ribose) polymerase (PARP), leading to apoptosis [74]. Ceramide also induces the intrinsic apoptosis pathway through the regulation of the liberation of cytochrome c from the disruption of ΔΨm [74].

In conclusion, this study provides evidence that high levels of FASN expression in cancer cells are crucial for cell proliferation and survival as well as for sensitivity to capsaicin or other FASN inhibitors. Capsaicin’s decreasing FASN expression and fatty acid synthesis is mediated by ROS generation which in turn triggers the dissipation of ΔΨm and the apoptotic pathway. The lipogenic enzyme FASN, not ACC and ACLY, is proposed to be the particular target of capsaicin to induce apoptosis in HepG2 cells. This study also suggests that an accumulation of malonyl-CoA as a result of a reduction of fatty acid synthesis is a critical pro-apoptotic factor that inhibits CPT-1 activity, leading to accumulation of ceramide which in turn induces apoptosis. This research offers new insight into capsaicin as an important anti-cancer agent, acting by inducing apoptosis through an inhibition of *de novo* fatty acid synthesis.
